# 762. Real-World Utilization of *C. difficile* Drug Treatments and Associated Clinical Outcomes in a US Hospital System

**DOI:** 10.1093/ofid/ofab466.959

**Published:** 2021-12-04

**Authors:** Lauren McDaniel, Nathan Everson, Melissa White, Engels N Obi, Yiyun Chen, Rose Kohinke, Ellen Rachel Lockhart

**Affiliations:** 1 Carilion Clinic, Roanoke, VA; 2 Lancaster General Health, Exton, Pennsylvania; 3 Merck & Co., Rahway, New Jersey; 4 Merck & Co., Inc, Rahway, New Jersey; 5 University of Pittsburgh Medical Center Pinnacle, Harrisburg, Pennsylvania

## Abstract

**Background:**

IDSA recommends use of fidaxomicin or oral vancomycin for treatment of initial episode or first recurrence of *Clostridioides difficile* infection (CDI). This study aimed to evaluate impact of a clinical decision support order set driving appropriate use of fidaxomicin on utilization of CDI drug treatments and associated clinical outcomes.

**Methods:**

This was a retrospective, quasi-experimental study evaluating CDI therapies pre- (8/2016-11/2017) and post- (5/2018-1/2020) CDI order set implementation at a level-one trauma center located in Virginia. Admitted adult patients were included if CDI testing was positive for a 1^st^ or 2^nd^ episode and received active CDI treatment. Exclusions included fulminant CDI and CDI diagnosis by PCR with < 3 bowel movements or laxative use within 24 hours. The primary outcome was CDI recurrence within 30 days of completing therapy in patients who achieved clinical cure. Secondary outcomes were evaluated at 30 and 90 days and included sustained response and CDI-related readmissions.

**Results:**

After screening, 186 patients in the pre-group and 187 in the post-group were included. Median age was 68 [59-77], most patients had an initial CDI episode (88.2%) and were diagnosed with severe CDI (50.7%). Baseline characteristics were similar between each group on Charlson comorbidity index, ICU admission, CDI risk factors, and concomitant antibiotic use. Primary treatment options in the pre-group were most commonly metronidazole 47.9% and oral vancomycin 50.5%, and in the post-group were fidaxomicin 56.7% and oral vancomycin 41.7% (Figure 1). CDI recurrence rates at 30 days post-index medication (17.2% vs. 6.3%, p=0.004) were lower in the post-group (Table 1). Clinical cure (84.4% vs. 94.1%, p=0.002) and sustained response at 90 days (55.9% vs. 73.3%, p< 0.001) were higher in the post-group. CDI recurrence rates at 90 days and CDI-related readmissions at 30 and 90 days were also lower in the post group.

Figure 1. CDI Treatment Utilization

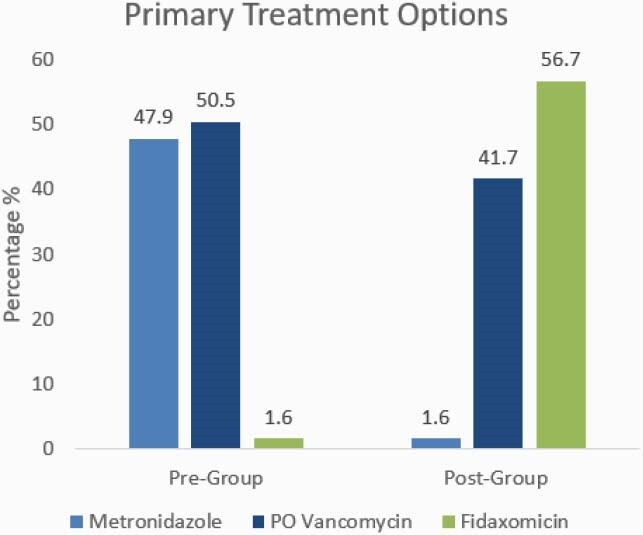

Table 1. Clinical Outcomes

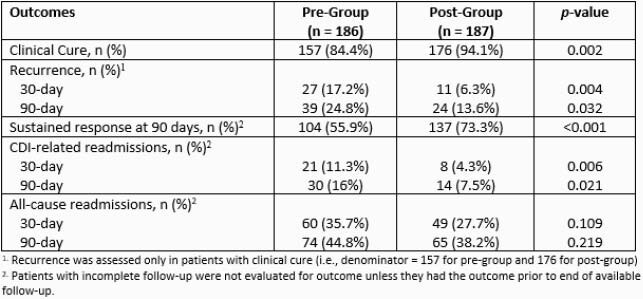

**Conclusion:**

Implementation of the CDI order set increased fidaxomicin use and was associated with a decrease in CDI recurrences and CDI-related readmissions and increase in clinical cure and sustained response. Findings suggest increased first-line use of fidaxomicin results in better clinical outcomes.

**Disclosures:**

**Lauren McDaniel, Pharm.D., BCIDP**, **Merck Sharp & Dohme Corp** (Grant/Research Support) **Nathan Everson, Pharm.D., BCIDP, AAHIVE**, **Merck & Co.** (Grant/Research Support) **Melissa White, PharmD**, **Merck Sharpe & Co** (Grant/Research Support) **Engels N. Obi, PhD**, **Merck & Co.** (Employee, Shareholder) **Yiyun Chen, PhD**, **Merck & Co., Inc** (Employee) **Rose Kohinke, PharmD**, **Merck Sharpe & Co** (Research Grant or Support)

